# Data on *Leptospira interrogans* sv *Pomona* infection in Meat Workers in New Zealand

**DOI:** 10.1016/j.dib.2017.05.053

**Published:** 2017-06-08

**Authors:** M. Pittavino, A. Dreyfus, C. Heuer, J. Benschop, P. Wilson, J. Collins-Emerson, P.R. Torgerson, R. Furrer

**Affiliations:** a Department of Mathematics, University of Zurich, Zurich, Switzerland; b Section of Epidemiology, Vetsuisse Faculty, University of Zurich, Zurich, Switzerland; c Institute of Veterinary Animal and Biomedical Sciences, Massey University, Palmerston North, New Zealand; d Department of Computational Science, University of Zurich, Zurich, Switzerland

**Keywords:** Leptospirosis, Interviews, Bayesian networks, Markov chain Monte Carlo, Bootstrapping

## Abstract

The data presented in this article are related to the research article entitled “Comparison between Generalized Linear Modelling and Additive Bayesian Network; Identification of Factors associated with the Incidence of Antibodies against *Leptospira interrogans* sv Pomona in Meat Workers in New Zealand” (Pittavino et al., 2017) [5].

A prospective cohort study was conducted in four sheep slaughtering abattoirs in New Zealand (NZ) (Dreyfus et al., 2015) [1]. Sera were collected twice a year from 384 meat workers and tested by Microscopic Agglutination for *Leptospira interrogans* sv Pomona (Pomona) infection, one of the most common *Leptospira* serovars in humans in NZ. This article provides an extended analysis of the data, illustrating the different steps of a multivariable (i.e. generalized linear model) and especially a multivariate tool based on additive Bayesian networks (ABN) modelling.

## **Specifications Table**

**Table:** 

Subject area	*Statistics and Epidemiology*
More specific subject area	*Applied Statistics, Graphical Modeling, Environmental and Working Exposure Factors*
Type of data	*Table, graph and figures*
How data was acquired	*The raw data was acquired by blood sampling and interviewing meat workers.* Sera was tested by *Microscopic Agglutination and values entered into an Access database. Data was analysed with GLM and ABN models.*
Data format	*Raw, Analyzed*
Experimental factors	*Voluntarily participating meat workers from four sheep abattoirs in the North Island of New Zealand*
Experimental features	*Work position, protective equipment usage, demographic variables and habits outside work were measured for the participants*
Data source location	*Palmerston North, New Zealand*
Data accessibility	*All data are available with this article at the public repository:*https://git.math.uzh.ch/reinhard.furrer/DIB-D-17-00344/tree/master

## **Value of the data**

• The data highlights exposure factors for Pomona infection for meat workers in sheep abattoirs.
• The data can be useful for other researchers investigating risk factors associated with *Leptospira* infection.
• The data provide an extended analysis on the usage of protective equipment when working in meat abattoirs.
• The data summarize important steps of a multivariate innovative approach called additive Bayesian network methodology.
• The data show the effect and advantages of working with graphical models, thanks to visual representation of the interconnection and correlation between all the variables analysed.

## 1 Data

**Fig. 1:**
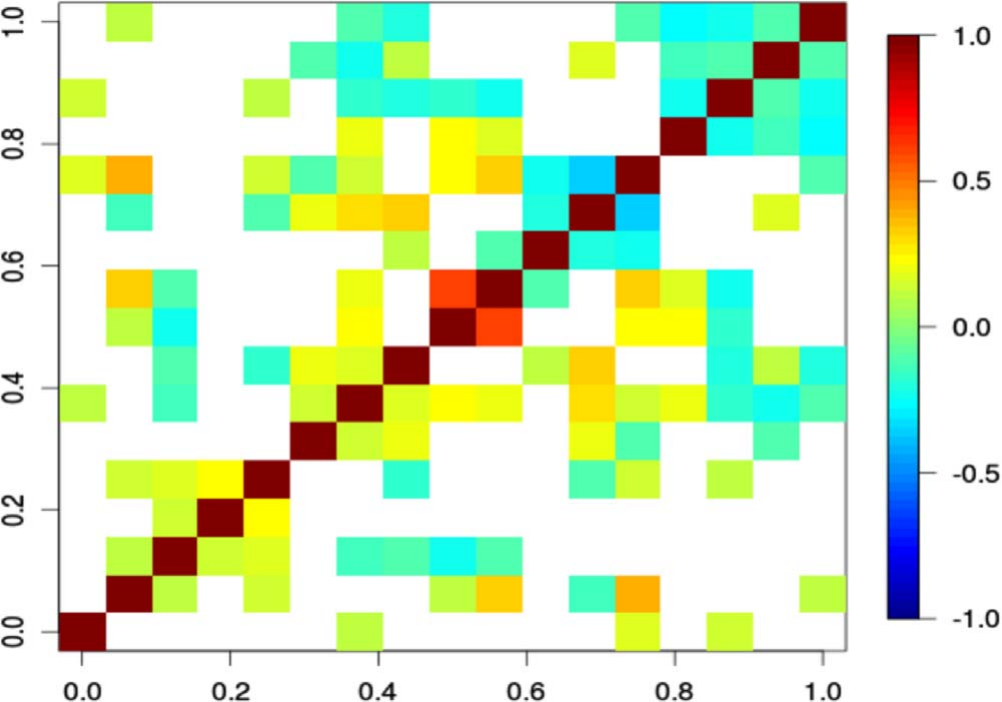
Resulting Spearman correlation׳s matrix between all the 17 variables in the dataset. The variables’ order is “Lepto”, “Sex”, “Hunt”, “Farm”, “Kill”, “Glov, “Glass, “Mask, “Age, “Time, “Work1, “Work2”, “Work3”,“Plant1”, “Plant2”, “Plant3” and “Plant4”. From this first exploratory data analysis looking at the first column and last row in the data set, we can see that the “Lepto” (Pomona infection) variable is mainly linked to variables “Work3” and “Plant2”, with an higher correlation (0.3) and with “Glass” with a smaller correlation (0.1).

The data were collected from 384 voluntarily participating meat workers from four purposively selected sheep abattoirs in the North Island of NZ. The outcome was “Pomona” infection in meat workers and the main exposure variables of interest were “work position”, the usage of “protective equipment” (PPE), “hunting”, “home slaughter” and “farming”. The total data set comprised of 17 variables with 15 binary and 2 continuous variables, listed in the descriptive Table 1 with their abbreviations used for the graphical model. The correlations between all these variables can be found in Fig. 1. Further variable names and their description can be found in Table 1 in [5], where the current variables “Lepto” and “Sex” have been respectively renamed “Pomona” and “Gender”. A detailed description of the protective equipment worn by sheep abattoir workers in each work position category can be found in Table 2. The number of “Pomona” infected workers, stratified by each working position and by the number of protected gear worn, is shown in Table 3. Data were extensively analysed with multivariable (i.e. GLM and GLMM) and multivariate techniques (i.e. ABN: additive Bayesian networks).

**Table 1:** Data of new infection with Leptospira interrogans sv Pomona in abattoir workers processing sheep in New Zealand: variable names and categories with their abbreviations used in the graphical model.

**Variables** and Categories in GLM and ABN model (Node label)
**Work position 0 (Work0^1^)**
0 Not working in boning, chillers, office
1 Working in boning, chillers, office
**Work position 1 (Work1)**
0 Not working in offal removal, pet food
1 Working in offal removal, pet food
**Work position 2 (Work2)**
0 Not removing intestines or kidneys, not inspecting meat
1 Intestines or kidney removal, meat inspection
**Work position 3 (Work3)**
0 Not working in yards, not stunning or pelting
1 Working in yards, stunning or pelting
**Abattoir 1 (A1)^1^**
0 Not working in Abattoir 1 (A1)
1 Working in Abattoir 1 (A1)
**Abattoir 1 (A2) (Plant1)**
0 Not working in Abattoir 1 (A2)
1 Working in Abattoir 1 (A2)
**Abattoir 2 (Plant2)**
0 Not working in Abattoir 2
1 Working in Abattoir 2
**Abattoir 3 (Plant3)**
0 Not working in Abattoir 3
1 Working in Abattoir 3
**Abattoir 4 (Plant4)**
0 Not working in Abattoir 4
1 Working in Abattoir 4
**Gender (Gender)**
0 Female
1 Male
**Hunter of goats, pigs & or deer (Hunt)**
0 No
1 Yes
**Slaughter of sheep, goats, pigs, beef & or deer at home (Kill)**
0 No
1 Yes
**Owning a farm with pigs, goats, sheep, beef cattle, alpaca & or deer (Farm)**
0 No
1 Yes
**Wearing normal or safety glasses (Glass)**
0 Sometimes/never
1 Always/often
**Wearing gloves on both hands (Gloves)**
0 Sometimes/never
1 Always/often
**Wearing a facemask (Mask)**
0 Sometimes/never
1 Always/often
**Months worked in the meat industry (Time)**
Continuous
**Age (Age)**
Continuous

^1^  Variable omitted from the analysis, in order to avoid over-parametrisation, due to transformation from categorical to binary variables.

**Table 2:** Type, number and percentage of protective equipment worn by sheep abattoir workers in each work position category for data of new infection with Leptospira interrogans sv Pomona in abattoir workers processing sheep in New Zealand.

**Work position**	**Facemask, N (%)**	**Gloves, N (%)**	**Safety Glasses, N (%)**	**Total**
**Work position 0 (Work0^1^)**				
1 Working in boning, chillers, office	1 (0.7)	81 (57.0)	40 (28.2)	142
**Work position 1 (Work1)**				
1 Working in offal removal, pet food	13 (29.5)	32 (72.7)	30 (68.2)	44
**Work position 2 (Work2)**				
1 Intestines or kidney removal, meat inspection	35 (39.8)	74 (84.0)	73 (82.9)	88
**Work position 3 (Work3)**				
1 Working in yards, stunning or pelting	15 (13.6)	63 (57.3)	75 (68.2)	110
Total	64 (16.7)	250 (65.1)	218 (56.8)	384

^1^  Variable omitted from the analysis, in order to avoid over-parametrisation, due to transformation from categorical to binary variables.

**Table 3:** Number of “Pomona” infected workers, stratified by working position and protective gear worn for data of new infection with Leptospira interrogans sv Pomona in abattoir workers processing sheep in New Zealand. In bold are reported “Pomona” cases corresponding to the overall population and not to subset related to specific conditions.

**Variables**	**N**
**Pomona cases**	**36**
**Pomona cases wearing all 3 protective equipment**	**5**
**Pomona cases wearing both glasses and gloves**	**21**
**Pomona cases wearing at least one of the 3 protective equipment**	**35**
**Pomona cases when wearing facemask in general and when working in various work positions**	
Lepto|Mask	**7**
Lepto|Mask,Work0	0
Lepto|Mask,Work1	0
Lepto|Mask,Work2	*4*
Lepto|Mask,Work3	*3*
**Pomona cases when wearing glasses in general and when working in various work positions**	
Lepto|Glass	**26**
Lepto|Glass, Work0	1
Lepto|Glass, Work1	4
Lepto|Glass, Work2	*11*
Lepto|Glass, Work3	*10*
**Pomona cases when wearing gloves in general and when working in various work positions**	
Lepto|Gloves	**28**
Lepto|Gloves, Work0	1
Lepto|Gloves, Work1	3
Lepto|Gloves, Work2	*10*
Lepto|Gloves, Work3	*14*

## 2 Experimental design, materials and methods

### 2.1 Experimental design

A prospective cohort study amongst 384 voluntarily participating meat workers from four purposively selected sheep abattoirs in the North Island of NZ was conducted. Study methods were described in detail by Dreyfus et al. [1], [5]. Participants were blood sampled and interviewed at the same time using a questionnaire [5]). Sera were collected twice a year and tested by Microscopic Agglutination for *Leptospira interrogans* sv Pomona infection. New infection occurred where a worker sero-converted or had an anamnestic response [1], [2], [3].

### 2.2 Data on GLM and GLMM

Data were analysed using the software R, version 3.1.2 [4]. Crude associations between the risk of infection with Pomona and potential risk, protective or confounding factors, listed in Table 1 in [5], were calculated by univariable analysis. We used a multivariable generalized linear model (GLM) to test for significant risk factors for new Pomona infection, adjusting for the effect of others (Table 2 in [5]). A multilevel generalized linear mixed model (GLMM) using abattoir as a random effect was also used, in order to evaluate the effect of clustering by abattoir on the model outcome, see Table 4.

**Table 4:** Odds ratio (OR), 95% confidence intervals (95% CI) and *p*-value of multivariable mixed effects logistic regression (GLMM – with abattoir as a random effect) assessing the association in meat workers between new infection with “Pomona” and the risk factors from the best fitting GLM model identified for data of new infection with Leptospira interrogans sv Pomona in abattoir workers processing sheep in New Zealand..

**Variables**	**OR**	**95% CI**	***P*-value**
Work1	20.6	2.2–195.8	0.01
Work2	29.7	3.7–239.3	0.00
Work3	50.7	6.1–421.2	0.00
Sex	0.53	0.2–1.38	0.19

### 2.3 Data on ABN model

**Fig. 2:**
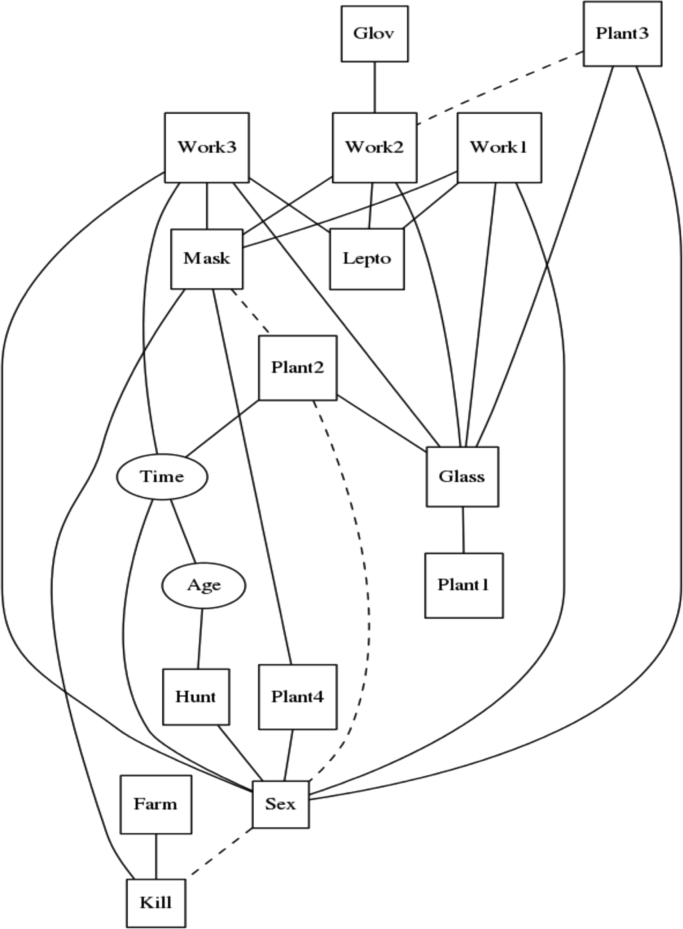
Optimal ABN model from the first step of ABN analysis, with a maximum number of seven parents, for data of new infection with *Leptospira interrogans* sv Pomona in abattoir workers processing sheep in New Zealand. Dashed lines represent the arcs not supported after the bootstrapping analysis.

**Fig. 3:**
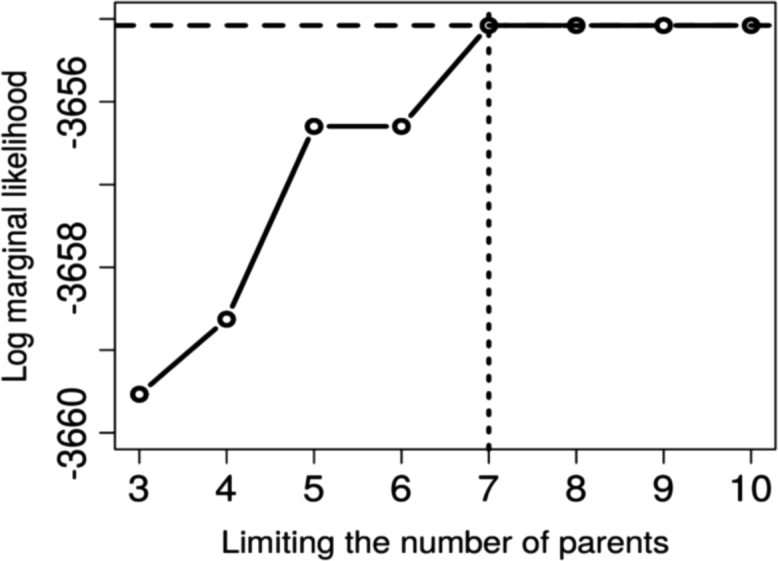
Comparison of goodness-of-fits (log marginal likelihood) for different parent limits (number of covariates in each regression model at each node), resulting from the first step of model selection in ABN methodology, for data of new infection with *Leptospira interrogans* sv Pomona in abattoir workers processing sheep in New Zealand.
(2) In the second step, the model was adjusted by checking it for over-fitting [10], [11] using Markov chain Monte Carlo (MCMC) simulation implemented in JAGS (‘just another Gibbs sampler’) [10], [11]. A visual check of the marginal densities estimated from the initial ABN model (Fig. 2) was conducted, in order to verify that the posterior densities integrate to one (Fig. 4). Simulated datasets were generated with MCMC as iterations of an identical size as the original one, from the optimal model found in step one. It was repeated 2560 times (Fig. 5), arcs not covered at least 50% (dashed lines in Fig. 2) were retrieved from the final globally optimal ABN.

All analyses were conducted using the R package “abn” [6]. A three-step procedure was utilized:

(1) The first step was to find an optimal model (Fig. 2), [7], [8], [9] using an order based exact search method [9]. The best goodness-of-fit to the available data was computed using the marginal likelihood method (Fig. 3) [6].

**Fig. 4:**
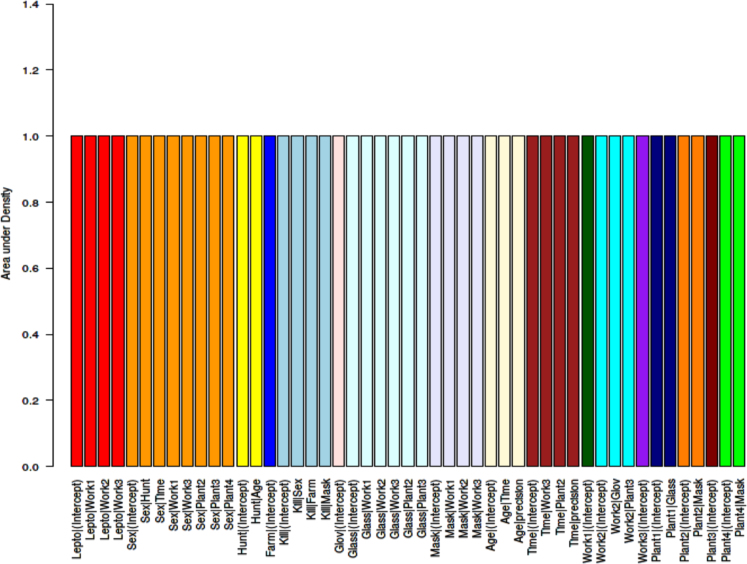
Approximate area under the marginal densities resulting from the ABN model identified at the first step of ABN methodology, for data of new infection with *Leptospira interrogans* sv Pomona in abattoir workers processing sheep in New Zealand.

**Fig. 5:**
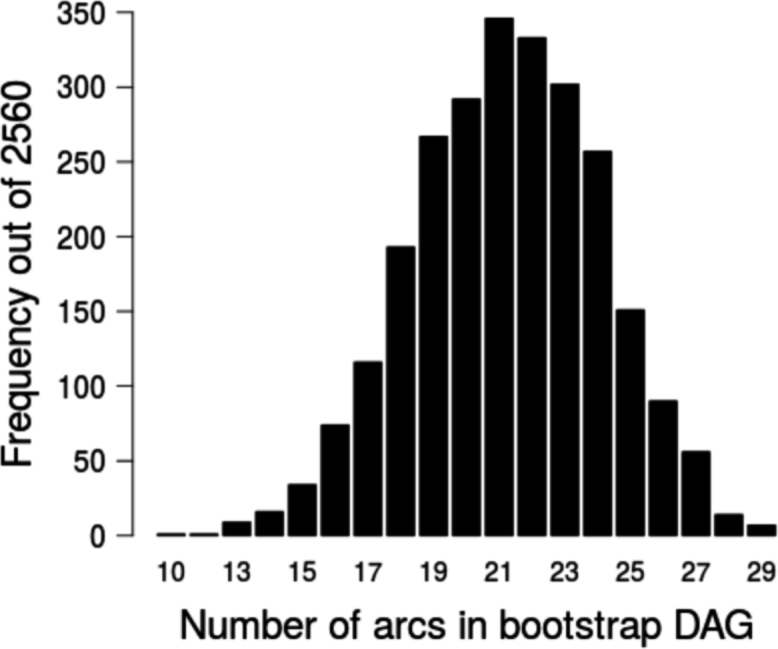
Number of arcs recovered in bootstrapping, resulting from the bootstrapping in ABN methodology, for data of new infection with *Leptospira interrogans* sv Pomona in abattoir workers processing sheep in New Zealand.
(3) In the third step of ABN analysis, the marginal posterior log odds ratio and 95% credible intervals were estimated for each parameter from the posterior distribution (Fig. 6), expressed by the ABN model identified at the second step (Fig. 1 in [5]).

**Fig. 6:**
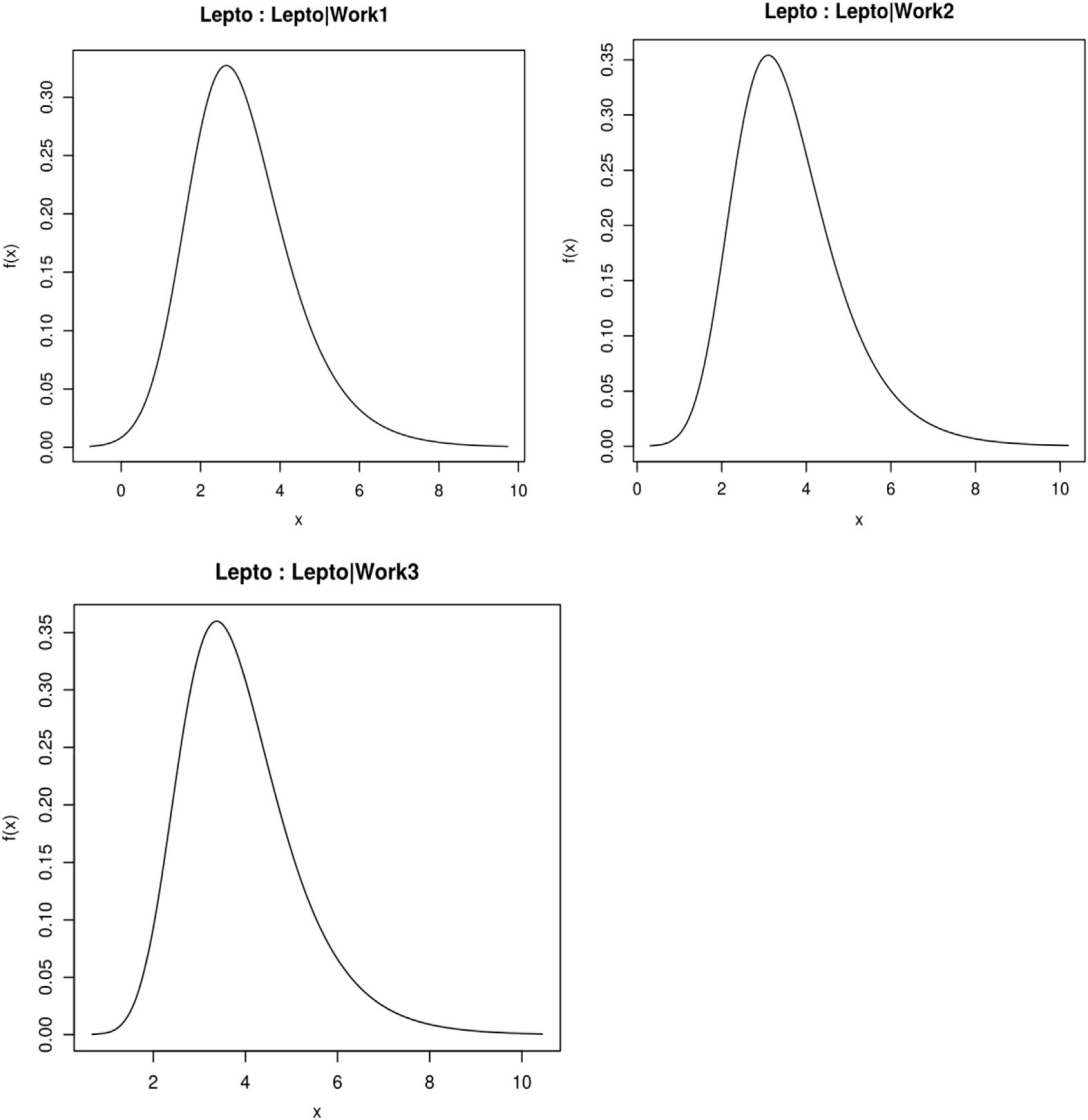
Selected posterior density plots from the final ABN model, related to the outcome variable of interest “Pomona” (“Lepto” in the figure) for data of new infection with *Leptospira interrogans* sv Pomona in abattoir workers processing sheep in New Zealand.
