# Supplementary material for: Data on Leptospira interrogans sv Pomona infection in Meat Workers in New Zealand
Source: Data Brief. 2017 Jun 8;13:587–96. doi: 10.1016/j.dib.2017.05.053 (PMC5496473; doi:10.1016/j.dib.2017.05.053)
Supplement: Supplementary file 1 — Supplementary material [file mmc1.pdf]

CONFLICT OF INTEREST FORM

Dear ‘Data in Brief’ Editors,

The authors declare that they have no competing interests.

Kind regards,

Marta Pittavino and Co-authors

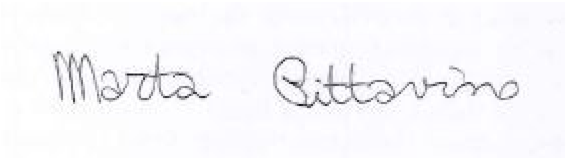

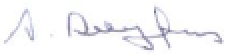

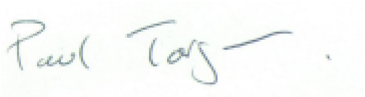

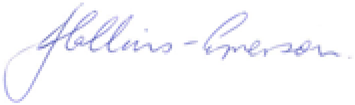

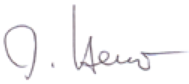

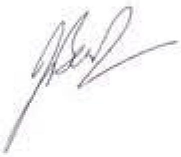

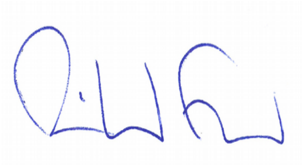

P R Wil[illegible]
